# Impaired folate status in patients with mental disorders

**DOI:** 10.1017/neu.2025.1

**Published:** 2025-01-20

**Authors:** Narvini Rajen, Hanne Wrengler Velure, Erik Johnsen, Anne-Lise Bjørke-Monsen

**Affiliations:** 1 Department of Medical Biochemistry and Pharmacology, Haukeland University Hospital, Bergen, Norway; 2 Division of Psychiatry, Haukeland University Hospital, Bergen, Norway; 3 Department of Clinical Medicine, University of Bergen, Bergen, Norway; 4 Mohn Research Center for Psychotic Disorders, Bergen, Norway; 5 Laboratory of Medical Biochemistry, Innlandet Hospital Trust, Lillehammer, Norway; 6 Laboratory of Medical Biochemistry, Førde Hospital Trust, Førde, Norway

**Keywords:** folate, cobalamin, homocysteine, mental disorders

## Abstract

**Objective:**

Folate and cobalamin deficiency or impaired function due to genetic variants in key enzymes have been associated with neuropsychiatric symptoms. The aim of this study was to compare folate and cobalamin status in patients admitted to an acute psychiatric unit to patients from primary health care in order to reveal factors which may be important in the follow-up of patients with mental disorders.

**Methods:**

Anonymous blood samples tested for folate, cobalamin, the metabolic marker total homocysteine (tHcy), creatinine and glomerular filtration rate as well as age and gender in patients admitted to a psychiatric acute unit (*n* = 981) and patients from primary health care (controls) (*n* = 32,201) were reviewed retrospectively.

**Results:**

Median serum folate was 18% lower and median serum cobalamin was 11% higher in patients with mental disorders compared to controls. Folate deficiency was associated with 54% higher median tHcy levels among patients with mental disorders compared to controls. The prevalence of folate deficiency was 31% and of cobalamin deficiency 6% in patients admitted to a psychiatric acute unit in a Norwegian hospital in 2024.

**Conclusion:**

Folate, but not cobalamin deficiency, was prevalent in Norwegian patients with mental disorders. The higher tHcy levels in folate-deficient patients with mental disorders indicate an impaired folate metabolism, which might be related to genetic factors, such as polymorphisms in the methylenetetrahydrofolate reductase (MTHFR) gene. Ensuring a serum folate concentration above 15 nmol/L and a serum cobalamin above 250 pmol/L might improve symptoms in patients with mental disorders.

## Significant outcomes

Folate, but not cobalamin, deficiency was common in patients admitted to an acute psychiatric unit

Patients with mental disorders had higher total homocysteine (tHcy) levels with folate deficiency than controls, something which might indicate a higher prevalence of genetic variants in the folate metabolism in this population

## Limitations

No genetic testing for the methylenetetrahydrofolate reductase (MTHFR) polymorphism was done

The control group included unselected patients from primary health care and could potentially include patient with mental disorders

As data were anonymous, it was not possible to control for potential medication interference with folate and cobalamin metabolism

## Introduction

Studies indicate that patients with mental disorders have a poor diet and an increased prevalence of vitamin insufficiency compared to the general population (Aucoin *et al*., 2020; Ljungberg *et al*., 2020; Gabriel *et al*., 2023). Essential micronutrients are vital to all cellular processes in the body, and particularly reduced levels of folate and cobalamin have been associated with neuropsychiatric symptoms and disease, including schizophrenia, bipolar disorder and major depression (Bottiglieri, 1996).

Folate in the form of 5-methyltetrahydrofolate and cobalamin in the form of methylcobalamin are necessary for remethylation of the amino acid homocysteine to methionine, an important methyldonor in metabolism (Selhub, 1999). In adults, total homocysteine (tHcy) is primarily a metabolic marker of folate status, but it is also a marker for cobalamin and to a lesser extent for vitamin B_6_ status and deficiency of either of these vitamins will increase tHcy concentrations (Schneede *et al*., 2000).

The enzyme methylenetetrahydrofolate reductase (MTHFR) transforms 5,10-methylenetetrahydrofolate to 5-methyltetrahydrofolate, the methyldonor in the remethylation of homocysteine to methionine (Bagley and Selhub, 1998). In the Nordic population, 5–8% have a polymorphism in the gene encoding for the 5,10-MTHFR (C677T, Ala --> Val) enzyme, late reductase (MTHFR) (C677T, Ala --> Val) enzyme, converting 5,10-methylenetetrahydrofolate to 5-methyltetrahydrofolate (Jacques *et al*., 1996). When serum folate levels are low, the MTHFR polymorphism will impair folate metabolism and the remethylation of homocysteine to methionine and thereby increase plasma tHcy levels (Savage *et al*., 1994; Green *et al*., 2017a).

The MTHFR polymorphism has been linked to an increased risk of bipolar disorder, schizophrenia and major depression in the overall population (Zhang *et al*., 2022).

As vitamin deficiencies and genetic variants may cause neurologic and psychiatric symptoms (Bottiglieri, 1996; Hutto, 1997), it is relevant to investigate vitamin status in patients with mental disorders. The aim of this study was to compare folate and cobalamin status in patients admitted to an acute psychiatric unit to patients from primary health care, in order to reveal factors which may be important in the follow-up of patents with mental disorders.

## Materials and methods

### Study population

The study group was patients with mental disorders admitted to the psychiatric acute unit at Haukeland University Hospital from January 1^st^ through December 31^st^ 2022 (*n* = 981). The routine test panel for these patients included serum cobalamin, plasma tHcy, serum creatinine including glomerular filtration rate (GFR), whereas serum folate was requested if indicated (in 100 of the 981 patients). In February 2024, serum folate was added to the routine test panel and test results from patients admitted to the psychiatric unit during the period from February 15 to October 15 2024 (*n* = 603) were included in order to find the prevalence of low folate and cobalamin status in patients with mental disorders.

The control group included unselected patients from the primary health care (controls), who had their serum folate, cobalamin and tHcy requested by their general practitioner (*n* = 32,201). All blood samples were analysed at the Department of Medical Biochemistry and Pharmacology at Haukeland University Hospital, Bergen, Norway, from January 1^st^ through December 31^st^ 2022.

The data on serum folate, serum cobalamin, plasma/serum tHcy, serum creatinine and estimated glomerular filtration rate (eGFR), in addition to gender and age were anonymously extracted from the laboratory data system. If a patient had multiple blood tests, only the first available test results from the defined study period were included. Patients <18 years were excluded in both groups. All controls (*n* = 32,201) and all admitted patients with mental disorders (*n* = 1584) had data on plasma/serum tHcy. Missing data in the control group included serum folate (*n* = 1693), serum cobalamin (*n* = 1582) and serum creatinine (*n* = 3884) and in the mental disorders admitted group was serum folate (*n* = 943), serum cobalamin (*n* = 3) and serum creatinine (*n* = 9).

The Regional Committee for Medical and Health Research Ethics, Region Western Norway, ref. number 2023/630455 evaluated and confirmed that the study ensured anonymity of the laboratory data and waived the need for informed consent.

### Biomarker analyses

The serum and plasma analyses were analysed in the routine accredited laboratory at Haukeland University Hospital, Bergen, Norway, on Roche Modular E and Cobas e 602 and Roche Modular P and Cobas c 702 (Roche Diagnostics, Basel, Switzerland). Serum folate and serum cobalamin were analysed with a competitive electrochemiluminescence immunoassay. Plasma and serum tHcy samples were measured using an enzymatic assay. Serum creatinine was analysed using an enzymatic reaction cascade with photometric detection. eGFR was automatically calculated with the CKD-EPI formula (Levey and Stevens, 2010). Analytical coefficient of variation was 5.7% for tHcy, 10% for folate, 7% for cobalamin and 3% for creatinine. The manufacturer states that no assay interference was found at therapeutic concentrations using common drug panels for folate, cobalamin or tHcy assays (Sonntag and Scholer, 2001).

The majority of the patients admitted with mental disorders had their tHcy measured in plasma, only 5% of the samples were analysed in serum. The opposite was true for the controls, as the majority of their tHcy samples were analysed in serum and only 0.5% in plasma.

The limit for quantification of folate was 1.4–45.4 nmol/L and for cobalamin 75–1476 pmol/L. For values below or above these limits, the numeric values for the limits of detection were used.

### Decision limits for folate and cobalamin deficiency

Plasma tHcy starts to increase when serum folate falls below ∼ 25–27 nmol/L, indicating suboptimal intracellular folate stores, and increases more sharply below ∼ 10 nmol/L, indicating biochemical deficiency (de Benoist, 2008; Chen *et al*., 2019; Bjørke-Monsen and Ueland, 2023). In adults with adequate folate status (serum folate >10 nmol/L), plasma tHcy start to increase when serum cobalamin falls below ∼500 pmol/L, indicating suboptimal intracellular cobalamin stores, with a steeper increase when serum cobalamin falls below 250 pmol/L indicating biochemical deficiency (Green *et al*., 2017b; Bjørke-Monsen and Lysne, 2023). Studies show that genomic instability in human cells is minimised when serum folate >25 nmol/L, serum cobalamin >300 pmol/L and plasma homocysteine <7.5 μmol/L in adults (Fenech, 2012; Fothergill *et al*., 2023), indicating that these figures represent a vitamin replete condition.

Based on changes in the metabolic marker tHcy, serum folate <10 nmol/L and serum cobalamin <250 pmol/L were used as decision limits for deficiency (de Benoist, 2008; Bjørke-Monsen and Lysne, 2023). Serum folate was categorised into Very low (< 5 nmol/L), Low (5–10 nmol/L), Normal (10–15 nmol/L) and High (≥ 15 nmol/L).

### Statistical analyses

The results are presented as median and interquartile range (IQR; 25^th^ and 75^th^ percentile) and compared using nonparametric Mann–Whitney *U* test. Categorical data was analysed using Pearson’s Chi-Square test. *P* values <0.05 was considered statistically significant. The data were analysed by the software “Statistical Package for the Social Sciences” (SPSS) version 29.

## Results

### Patient characteristics

Patient demographics are given in Table 1. Admitted patients with mental disorders were significantly younger, with a higher proportion of men compared to the control group (Table 1). Significant differences between the two groups were seen for all tested biochemical parameters (Table 1). Median serum creatinine was slightly higher, but median eGFR was lower in the admitted mental disorders group compared to the controls, possibly due to higher percentage of men and a lower median age among the admitted patients with mental disorders compared to the controls.

**Table 1.** Demographics and biochemical parameters for patients admitted with mental disorders (*n* = 981) and patients from primary health care (Controls, *n* = 32,201)

Parameters, median (25^th^–75^th^ percentile)	Patients with mental disorders *N* = 981	Controls *N* = 32,201	*P*-value
Age, years	39 (28–54)	49 (33–65)	<0.001^1^
Male gender, number (%)	558 (57%)	11,877 (37%)	<0.001^2^
Serum folate, nmol/L^3^	11.9 (7.2–19.8)	14.5 (9.7–24.1)	<0.001^1^
Serum cobalamin, pmol/L^4^	405 (311–543)	365 (287–473)	<0.001^1^
Plasma / Serum total homocysteine, µmol/L	11.8 (9.4–15.1)	11.4 (9.4–14.0)	<0.001^1^
Serum creatinine, µmol/L^5^	73 (63–83)	71 (62–81)	0.002^1^
eGFR, ml/min/1,73m^2,5^	103 (89–115)	95 (81–108)	<0.001^1^
eGFR <60, number (%)	37 (4%)	1590 (6%)	0.014^2^

1 Mann–Whitney *U* test.

2 Pearson’s Chi-Square test.

3 Serum folate data were available for *n* = 100 patients with mental disorders and *n* = 30,508 outpatients.

4 Serum cobalamin data were available for *n* = 978 patients with mental disorders and *n* = 30,619 outpatients.

5 Serum creatinine and estimated glomerular filtration rate (eGFR) data were available for *n* = 976 patients with mental disorders and *n* = 28,317 outpatients.

### Folate status

**Figure 1.**
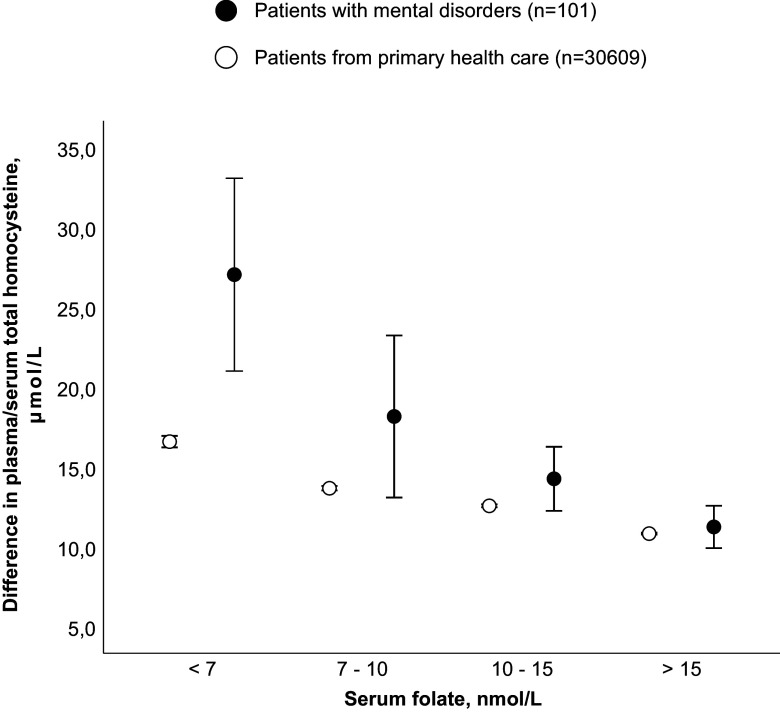
Total homocysteine (mean and 95% confidence interval) from patients admitted with mental disorders (plasma tHcy) and patients from primary health care (serum tHcy) in relation to folate categories.

In 2022 serum folate was requested in 10% of the patients admitted to the psychiatric acute unit. Their median serum folate level was 18% lower (Table 1) and a significantly higher percentage (43%) had folate deficiency (<10 nmol/L) compared to 27% of the controls (*p* < 0.001) (Table 2). Median tHcy concentration in admitted patients with mental disorders was 69% higher in the Very low folate group and 30% higher in the Low folate group compared to the controls (*p* < 0.001) (Table 2, Fig. 1).

**Table 2.** Demographics and biochemical parameters according to serum folate categories for patients admitted with mental disorders (*n* = 100) and patients from primary health care (Controls, *n* = 30,508)

Parameters, median (25^th^–75^th^ percentile)	Serum folate							
	<5 nmol/L		5–10 nmol/L		10–15 nmol/L		≥15 nmol/L	
	Patients with mental disorders	Controls	Patients with mental disorders	Controls	Patients with mental disorders	Controls	Patients with mental disorders	Controls
Number (%)	14 (14%)	1223 (4%)	30 (30%)	6888 (23%)	24 (24%)	7793 (26%)	33 (33%)	14,604 (48%)
Age, years	33 (28–45)	34 (25–48)	39 (30–52)	43 (29–59)	43 (26–56)	49 (34–65)	47 (31–58)	53 (36–68)
Male gender (%)	62%	39%	70%	41%	38%	40%	58%	33%
Plasma/serum total homocysteine, µmol/L	24.7 (16.3–43.0)	14.6 (11.8–20.0)	16.9 (13.0–23.5)	13.0 (10.8–15.9)	13.8 (10.3–17.2)	11.8 (10.0–14.3)	10.2 (9.2–12.3)	10.3 (8.5–12.5)
Serum cobalamin, pmol/L	338 (219–374)	323 (253–429)	325 (240–446)	346 (277–443)	325 (240–446)	346 (277–443)	435 (233–625)	394 (310–519
Serum creatinine, µmol/L	74 (63–82)	70 (63–80)	76 (67–90)	72 (64–82)	73 (61–80)	71 (63–82)	69 (61–79)	70 (62–81)
eGFR, mL/min/1,73m^2^	107 (102–115)	105 (91–117)	101 (88–114)	98 (85–111)	100 (77–120)	95 (82–108)	102 (86–118)	92 (79–106)

No differences were observed for serum cobalamin levels according to folate categories (*p* > 0.5) and apart from a significantly higher eGFR in patients admitted with mental disorders with High serum folate, there were no significant differences in renal function parameters between admitted patients with mental disorders and controls in each of the four folate categories (*p* > 0.08) (Table 2).

The prevalence of folate deficiency was 31% based on routine test panels performed in patients admitted with mental disorders in 2024.

### Cobalamin status

Median serum cobalamin level was 11 % higher in patients admitted with mental disorders compared to the controls. Cobalamin deficiency (<250 pmol/L) was seen in 14% in the controls. The prevalence of cobalamin deficiency was 11% in 2022 and 6% in 2024 in patients admitted with mental disorders based on routine test panels.

## Discussion

Median serum folate was 18% lower and median serum cobalamin was 11% higher in patients admitted with mental disorders compared to controls from primary health care. Folate deficiency was associated with higher tHcy levels among patients admitted with mental disorders compared to the controls.

The prevalence of folate deficiency was 31% and of cobalamin deficiency 6% in patients admitted to a psychiatric unit in a Norwegian hospital in 2024.

The definition of low folate and cobalamin levels differ substantially between studies (Green *et al*., 2017b; Bjørke-Monsen and Lysne, 2023; Bjørke-Monsen and Ueland, 2023), and this must be taken into account when prevalence patterns are discussed. Depending on the chosen cut-off, the prevalence of folate deficiency among psychiatric inpatients is reported to be 10–33%, and the prevalence of cobalamin deficiency 5–30% (Skerritt, 1998; Silver, 2000). The chosen definition in our study was below 10 nmol/L for serum folate and below 250 pmol/L for serum cobalamin deficiency and based on this, the prevalence for folate deficiency was 31% and for cobalamin deficiency 6% in patients admitted to a psychiatric unit in 2024. The prevalence for cobalamin deficiency was reduced from 11% in 2022 to 6% 2024, something which might be due to an increased awareness of the importance of adequate vitamin status, both among patients and health professionals during this period.

Low B vitamin levels have repeatedly been linked to mental disorders, particularly depression (Skerritt, 1998; Lerner *et al*., 2006; Clement *et al*., 2007; Liwinski and Lang, 2023). Our patients admitted with mental disorders had lower serum folate, but higher median serum cobalamin compared to the controls. B-vitamin status is mainly a function of diet (Allen, 2008), and patients with serious mental disorders are on the group level reported to have a poor diet characterised by a low consumption of fibre and fruit (Dipasquale *et al*., 2013). An inverse association is also reported between intake of fruits and the likelihood of severe depression, anxiety and *psychological* distress symptoms (Shams-Rad *et al*., 2022). A low intake of fruits will reduce serum folate, but not serum cobalamin, a vitamin only found in animal foods.

The prevalence of cobalamin deficiency was lower (6%) among patients admitted with mental disorders, compared to 14% in requested test in controls from primary health care. One would expect requested tests to have a higher prevalence of vitamin deficiency. In addition, the prevalence of cobalamin deficiency is reported to increase with age (Green *et al*., 2017b), and the controls were older than patients admitted with mental disorders. A 6% prevalence of cobalamin deficiency was found in young women based on a serum cobalamin <220 pmol/L (Al-Musharaf *et al*., 2020) compared to 14.5% in a population of outpatients above 65 years (Pennypacker *et al*., 1992).

Among patients with folate deficiency, the median tHcy level was 54% higher in patients admitted with mental disorders compared to controls. Several factors known to affect tHcy concentrations might explain this observation (Schneede *et al.,* 2000).

Both folate and cobalamin deficiency impair homocysteine remethylation and increase plasma tHcy levels (Schneede *et al.,* 2000). We did not measure vitamin B_6_ status, but plasma tHcy is reported to be more strongly influenced by folate and vitamin B_12_ than vitamin B_6_ status and moderate B_6_ deficiency is reported to cause only a mild increase in plasma tHcy concentration (Green *et al*., 2017b).

The tHcy concentration tend to increase with age and reduced renal function (Schneede *et al.,* 2000). However, the patients admitted with mental disorders tended to be younger than the controls, and there were no significant differences in renal function parameters between the groups in each of the four folate categories.

Patients admitted with mental disorders included more men than the controls and as tHcy concentrations are reported to be higher in men than in women, this might be relevant for the observed higher tHcy concentrations in patients admitted with mental disorders. The tHcy difference between the genders is however not large, the reported geometric mean was 14.8 (SD 6.2) µmol/L in adult men compared to 13.4 (SD 4.8) µmol/L in adult women (de Bree *et al*., 2001), indicating a 10% difference between men and women.

In addition, some medications, such as valproate and lamotrigine, commonly used mood stabilisers for the treatment of bipolar disorder, can potentially interfere with folate metabolism, thereby increasing tHcy concentrations (Baek *et al*., 2013). As this study was based on anonymous data, we were unable to control for medications.

Preanalytical factors may have an impact on tHcy levels. Due to local laboratory routines, tHcy was measured in EDTA-plasma for 95% of the admitted patients with mental disorders, and in serum for 99.5% of the controls from primary health care. The concentration of tHcy in whole blood increases at room temperature, because of a continuous production and release of homocysteine from blood cells. The artificial increase is reduced if the blood sample is placed on ice (Ueland *et al*., 1993). In the hospital laboratory, the EDTA sample is placed in ice-water and plasma is separated after maximum two hours. This optimal handling of the blood sample results in lower tHcy concentrations in plasma compared to serum (Vester and Rasmussen, 1991; Ueland *et al*., 1993; Pfeiffer *et al*., 2000). So according to preanalytical factors, one might expect the tHcy concentration to be lower in patients admitted with mental disorders compared to controls, not the opposite as we observed.

We did only observe a difference in tHcy concentration between the two groups when serum folate concentrations were low. The MTHFR polymorphism is the most common genetic cause of increased tHcy concentrations, but it only affects tHcy levels if serum folate is low (Bagley and Selhub, 1998; Liew and Gupta, 2015). Individuals with a homozygous MTHFR polymorphism are therefore recommended to maintain a serum folate level >15 nmol/L to improve folate metabolism (Huang *et al*., 2018).

In our study low folate concentrations were more common among patients admitted with mental disorders compared to controls from primary health care. Among requested blood tests in 2022, folate deficiency was found in 43% of patients admitted with mental disorders, which was significantly more compared to the 27% observed in the controls. This can be due to a poorer diet among patients admitted with mental disorders, however, the C677T MTHFR polymorphism is also per se associated with lower serum folate concentrations (Nishio *et al*., 2008).

Our data indicate that folate deficiency might have a greater impact on homocysteine metabolism in some patients with mental disorders, and this might be related to genetic variants, such as polymorphisms in the MTHFR gene. This hypothesis is supported by several publications reporting significant associations between the C677T polymorphisms and mental disease, like schizophrenia, major depression and bipolar disorder (Sazci *et al*., 2005; Gilbody *et al*., 2007, Peerbooms *et al*., 2011, Zhang *et al*., 2022). We did however not do any genetic testing for MTHFR polymorphism among patients with mental disorders, which is a major limitation of the study.

Folate and cobalamin play crucial roles in central nervous system metabolism, and deficiency may cause neurologic and psychiatric symptoms (Bottiglieri, 1996; Hutto, 1997). Optimising vitamin status by ensuring that patients have a serum folate above 15 nmol/L and a serum cobalamin above 250 pmol/L may improve psychiatric symptoms (Roffman *et al*., 2013; Allott *et al*., 2019; Lam *et al*., 2022).

## Conclusion

Folate deficiency is more prevalent than cobalamin deficiency in Norwegian patients admitted with mental disorders. Among patients with folate deficiency, patients admitted with mental disorders have significantly higher tHcy concentrations compared to unselected patients from primary health care, indicating an impaired folate metabolism, which might be related to polymorphisms in the MTHFR gene. Optimising vitamin status, ensuring a serum folate above 15 nmol/L and serum cobalamin above 250 pmol/L, is recommended for patients with mental disorders.
